# Beer Drinking

**Published:** 1854-11

**Authors:** 


### BEER DRINKING.

I HAVE been repeatedly asked whether drinking Porter would not be a benefit in certain cases of debility, loss of flesh, and the like. I have never advised the use of Beer, Porter, Ale, or anything of that kind, as a medicine, or for any other purpose, because they do not give permanent and substantial advantages. A person using these articles will undoubtedly in numerous instances "*fatten up*," at least in appearance, especially in the fall, but it is a deceptive gain; up to a certain point, there is an apparent improvement from its use, but all at once that improvement ceases, and the person sinks rapidly to the grave. There is a point at which it loses all its power, and, when it ceases to afford its accustomed stimulus, the person dies, precisely as in the habitual and excessive use of Brandy, or Tobacco, or Opium.

"Medical men," says Dr. GORDEN, "are familiar with the fact that beer drinkers in London can scarcely scratch their fingers without risk of their lives. A copious London beer drinker is one vital part. He wears his heart on his sleeve; bare to a death wound even from a rusty nail, or the claw of a cat." SIR ASTLEY COOPER, on one occasion, was called to a drayman who had received an injury in his finger from a small splinter of a stone. Suppuration had taken place. This distinguished surgeon opened the small abscess with his lancet. On returning, he discovered that he had forgotten his lancet case; going for this he found his patient in a dying state. "Every medical man in London," concludes the same writer, "dreads, above all things, a beer drinker for a patient."

